# Revisiting an Expression Dataset of Discordant Inflammatory Bowel Disease Twin Pairs Using a Mutation Burden Test Reveals *CYP2C18* as a Novel Marker

**DOI:** 10.3389/fgene.2021.680125

**Published:** 2021-06-15

**Authors:** Juan Du, Jie Yin, Haojie Du, Jiawei Zhang

**Affiliations:** ^1^Department of Gastroenterology, First Affiliated Hospital of Zhejiang University School of Medicine, Hangzhou, China; ^2^Cancer Institute (Key Laboratory of Cancer Prevention and Intervention, China National Ministry of Education), Second Affiliated Hospital, Zhejiang University School of Medicine, Hangzhou, China; ^3^Department of Human Genetics, Zhejiang University School of Medicine, Institute of Genetics, Zhejiang University, Hangzhou, China

**Keywords:** inflammatory bowel diseases, ulcerative colitis, novel marker, CYP2C18, LOXL2

## Abstract

The aim of this study was to investigate the expression features of discordant inflammatory bowel disease (IBD) twin pairs to identify novel molecular features and markers. We collected an expression dataset of discordant twin pairs with ulcerative colitis and performed integrative analysis to identify the genetic-independent expression features. Through deconvolution of the immune cell populations and tissue expression specificity, we refined the candidate genes for susceptibility to ulcerative colitis. We found that dysregulated immune systems and NOD-related pathways were enriched in the expression network of the discordant IBD twin pairs. Among the identified factors were significantly increased proportions of immune cells, including megakaryocytes, neutrophils, natural killer T cells, and lymphatic endothelial cells. The differentially expressed genes were significantly enriched in a gene set associated with cortical and medullary thymocytes. Finally, by combining these expression features with genetic resources, we identified some candidate genes with potential to serve as novel markers of ulcerative colitis, such as CYP2C18. Ulcerative colitis is a subtype of inflammatory bowel disease and a polygenic disorder. Through integrative analysis, we identified some genes, such as CYP2C18, that are involved in the pathogenesis of IBD as well as some candidate therapeutic targets, such as LOXL2.

## Introduction

Inflammatory bowel disease (IBD) is a multifactorial disorder that includes three main subtypes, Crohn’s disease (CD), Ulcerative Colitis (UC), and IBD unclassified (IBDU) (Uhlig and Muise, 2017). The phenotypes of these disorders are usually caused by defects in the epithelial barrier and dysregulation in the innate and adaptive immune responses. The pathogenic pathways of IBDs overlap with those of other disorders, such as psoriasis (Parkes et al., 2013). Therefore, the underlying genetic or epigenetic factors likely have pleiotropy phenotype which further complicates the identification of the molecular etiology of IBDs.

Familial aggregation and twin-paired studies have provided evidence that genetic factors contribute to the pathogenesis of IBDs. For instance, concordance in the diagnosis of UC (15.3 vs. 3%) and CD (51.6 vs. 5.1%) is higher in monozygotic twins than dizygotic twins (Annese, 2020). The heritability of IBDs varies across subtypes, as has been observed in twin studies of UC and CD. Rare variants in IL10 and IL10RB have been shown to be highly penetrant for intestinal inflammation (Uhlig and Schwerd, 2016). However, the penetrance of some variants might be variable, and researches on rare variants in IBDs is limited. Complex phenotypes are observed in IBDs, and the genetic results support this complexity. Thus far, more than 240 genes or loci have been linked to the IBDs (Annese, 2020), which indicates that these disorders are polygenic. Genome-wide association studies have refined the candidate genes involved in the pathogenesis of IBDs and have identified candidates in the NOD2-related microbe sensing pathway (Jostins et al., 2012), IL-23 driven T-helper cell response (Liu et al., 2015), and autophagy (Schwerd et al., 2017).

IBDs are multifactorial disorders. Research on discordant twin pairs with IBDs has indicated some genetic-independent factors underlying the pathogenesis. For instance, differences were observed in the gut microbiome of the inflamed and non-inflamed segments of the gut in both CD and UC (Ryan et al., 2020). However, a recent report showed that the gut microbiome of the healthy cotwins from discordant IBD twin pairs displays IBD-like signatures, indicating that the discordant monozygotic twins with IBD not only share the same genetic background but also the same gut microbiome (Brand et al., 2021). Thus, the causative role of the gut microbiome in the pathogenesis of IBDs requires longitudinal follow-up to confirm. Based on these findings, we supposed that the differential expression patterns between the affected and unaffected discordant twins could provide clues to the mechanism underlying the pathogenesis of IBD. In this study, we identified dysregulation of seven immune cell subpopulations as factors that might contribute to IBD, especially those related to the cortical and medullary thymocyte. In addition, we identified some candidate makers and therapeutic targets for UC.

## Materials and Methods

### Microarray Dataset

We download a transcriptome dataset from the Gene Expression Omnibus (GEO) database that included 10 paired discordant monozygotic twins for UC (GSE22619). We also collected expression data from 20 paired unrelated healthy twins (GSE7821).

### Identification of Differentially Expressed Genes

We performed *rma* normalization of the gene array dataset of twin pairs and identified the Differential Express Genes (DEGs) using the *oligo* (Carvalho and Irizarry, 2010) and *limma* R packages (Ritchie et al., 2015). The cutoff for DEGs was a log fold change (| logFC|) > 2 and a *p*-value less than 0.01.

### Principal Component Analysis and Sample Similarity Analysis

We performed principal component analysis (PCA) on the expression dataset using the R package *FactoMineR* (Lê et al., 2008) and *factoextra*^1^.

### Gene Set Enrichment Analysis

We performed a Gene Ontology (GO) analysis and Kyoto Encyclopedia of Genes and Genomes (KEGG) enrichment using the R package *clusterProfiler* (Yu et al., 2012) and Gene Set Enrichment Analysis (GSEA) to evaluate the underlying expression spectrum of UC using the R package *GSEABase* (Morgan et al., 2020).

### Deciphering the Immune Cell Populations Using the Expression Dataset

We applied the expression matrix to xCell (Aran et al., 2017) to decipher the immune cell proportion with the parameters set as default and applied paired Wilcoxon.test to evaluate the differential cell populations.

### Deconvolution of the Tissue Expression Specificity of the DEGs

We downloaded the normalized RNA-seq dataset from Genotype-Tissue Expression (GTEx) Portal and tissue origin annotation information and used the t-distributed stochastic neighbor embedding (t-SNE) approach to investigate the tissue expression specificity of the identified DEGs and normalized the mean expression for each type of tissues. To keep the consistency of the gene names, the gene symbols were converted to Ensembl gene id by *gprofiler* (Reimand et al., 2007), and the genes unmatched were ruled out in the downstream analysis.

### Cell Culture and Treatments

The human colon cancer cell lines HCT116 and HT29 were used in this study Cell lines were cultured at 37°C under 5% CO_2_ in McCoy’s 5A (Gibco) supplemented with 10% (v/v) fetal bovine serum (FBS, Gibco) and penicillin G (100 U/ml, Gibco) and streptomycin (100 μg/ml, Gibco). To induce the inflammation status, the cell lines were stimulated with TNF-α (100 ng/ml) for 24 h. And the control group, the cell lines were treated with DMSO. All the experiments were repeated three times.

### Determine the Expression of CYP2C18

The expression level of *CYP2C18* was determined in cell lines, HCT116, HT29, and the control cell line. Cells were washed with ice-cold phosphate-buffered saline (PBS), harvested with a cell scraper (Corning), centrifuged at 2,000 rpm for 5 min at 4°C, and resuspend with RIPA buffer supplemented with PMSF (Sigma), protease inhibitor (Roche). Lysates were subjected to sonication and spun at 13,300 rpm at 4°C. The supernatant was transferred into new tubes. Protein concentrations were qualified with the BCA reagent (Thermo Scientific). Protein lysates were resolved on SDS-PAGE gels, and protein of interest was detected with one of the following antibodies: CYP2C18 (PA5-112394, Invitrogen), β-actin (ab8226, Abcam). Paraffin section from 7 unrelated UC affected cases and 5 normal controls were applied to immunohistochemistry, which was performed as recommended with the antibody CYP2C18 (PA5-112394, Invitrogen).

### Curation the Association of Variants in Given Candidate Genes With Risk of IBDs

The rare non-silent variants in the identified genes were further inspected based on the IBD Exome Browser^2^ following the guidelines as described before (Richards et al., 2015; Yin et al., 2019).

## Results

### Revisiting the Potential Causative Genetic Factors of IBDs

To evaluate the genetic architecture underlying IBDs, we retrieved candidate genes by text mining and estimate their prevalence and penetrance using the IBD exome browser. Second, we focused on high confidence variants likely to be disease causative based on the results of a previous fine-mapping study (Huang et al., 2017). Of the fine-mapped genes with at least one variant located in the coding region, six genes, *NOD2* (*n* = 7), *IL23R* (*n* = 3), *CCDC71* (*n* = 2), *BANK1* (*n* = 2), *MST1* (*n* = 2), and *FUT2* (*n* = 2) had recurring non-silent variants. We evaluated the potential phenotypes of IBD associated with these fine-mapped candidate genes, and we found that the terms abnormal immunological process, colitis, and inflammation of gastrointestinal or intestinal recurred in the top 10 human phenotypes (Supplementary Table 1). To validate this result, we evaluated the enriched non-silent variants in these candidate genes using the IBD exome browser (IBD Exomes Portal, 2021), and only *NOD2* was highly enriched with potential disease-causing variants (Data not shown). Although Mendelian IBD has been reported, the underlying genetic factors interact with other non-genetic factors to determine the final phenotype. Therefore, it might be difficult to identify all the penetrant variants for IBDs and combine multiple sources of dataset, including gene mutation burden test results, gene tissue expression specificity and differential expression genes between affected cases and control could refine the candidate genes (Supplementary Figure 1).

### Expression Features Identified in the Discordant Twin Pairs

**FIGURE 1:**
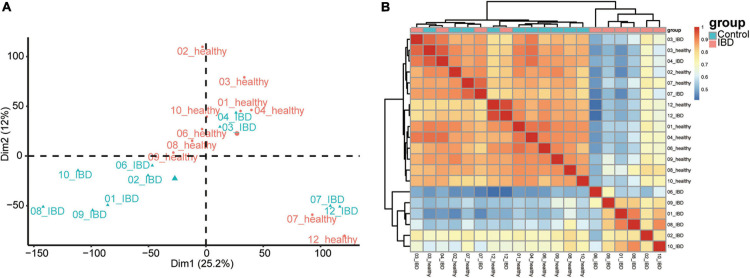
Sample inherent similarity based on principle component analysis (PCA) and pairwise correlation analysis. **(A)** Scatter plot of the top two components from the PCA. **(B)** Sample similarity derived from the pairwise correlation analysis. Pearson’s correlation results were shown in the heatmap.

To identify the most informative gene for distinguishing the affected twins from the unaffected twins, we retrieved the expression profile of discordant twin pairs (monozygotic) for IBDs (*n* = 10). We also included healthy twin pairs (*n* = 20) for quality control (Supplementary Figure 2). We first performed PCA on the healthy twin pairs cohort, and little distinction was observed among the individuals from each twin pairs (Supplementary Figure 2). In the discordant twin pairs for IBDs, the affected twins could be distinguished from the unaffected twins; however, in four twin pairs (ID: 03, 04, 07, and 12), there was little difference between the affected and unaffected twins (Figure 1A). We also performed a pairwise correlation using the expression dataset and obtained results similar to the PCA (Figure 1B). Although heterogeneity exists among different twin pairs, the distinction between the discordant pairs could provide specific information regarding the pathogenesis of IBD.

**FIGURE 2:**
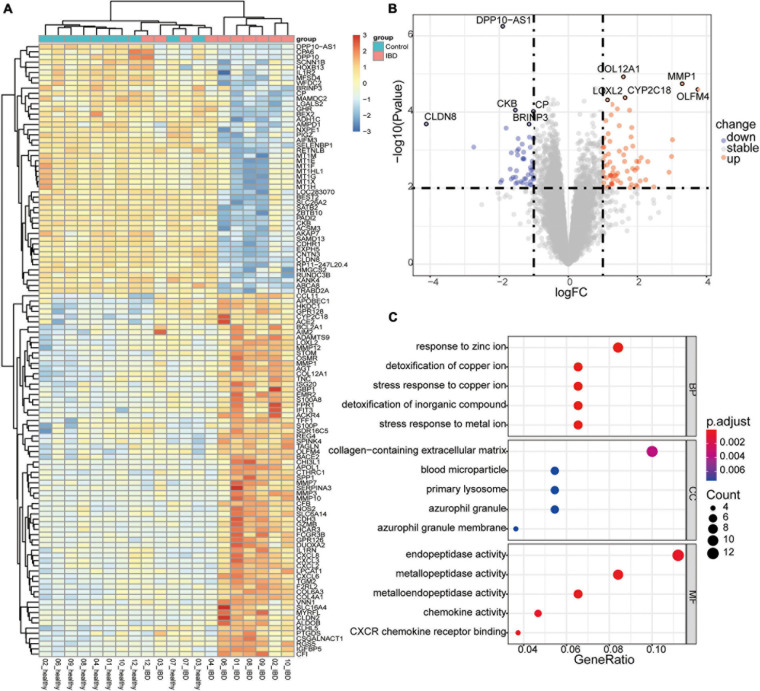
Differentially expressed genes (DEGs) in the discordant inflammatory bowel disease (IBD) twin pairs. **(A)** Heatmap of the scaled expression values of DEGs. **(B)** Volcano plot of the DEGs, with the top five upregulated and downregulated genes labeled. **(C)** Enriched components containing the DEGs.

To evaluate the expression dataset for possible noise, we first identify the DEGs among the healthy twin pair, and we did not identify any genes with significant differences (Supplementary Figure 3). Second, we examined the expression datasets from discordant IBD twin pairs. In total, we identified 118 genes that tended to be differentially expressed between the affected and unaffected twins (*P* < 0.01 and | log_2_FC| > 1; Figure 2A and Supplementary Table 2). A comparison to genes reported to be related to IBDs based on GWAS and DiseaseNet identified four genes, *CDH3*, *CXCL8*, *IL1R2*, and *NXPE1*, that were differentially expressed between the discordant twin pairs (Supplementary Figure 4 and Supplementary Table 3). Among the top up-regulated DEGs, *OLFM4*, *MMP1*, *CYP2C18*, *COL12A1*, and *LOXL2*, gene *OLFM4* encodes Olfactomedin4, which is a stem cell marker for small intestinal. The expression of *OLFM4* was highly upregulated in inflamed colonic epithelium, especially in UC (Gersemann et al., 2012; Suzuki et al., 2018). Furthermore, the relationships between IBD and some of the top DEGs (Figure 2B), such as *MMP1* (Wang and Qiu, 2010) and *COL12A1* (Ji et al., 2020), have also been reported. Interestingly, the serum levels of LOXL2 are significantly higher in primary sclerosing cholangitis (PSC) (Pollheimer et al., 2018), which is closely associated with IBD, and LOXL2 targeting in IBD has been evaluated in PSC (Lynch et al., 2019). Meanwhile, among the top down-regulated DEGs, such as *BRINP3*, *CKB*, *CP*, *CLDN8* and *DPP10-AS1*, down regulation of *BRINP3* (Smith et al., 2014), *CLDN8* (Wang et al., 2016), has been reported in UC. CLDN8 has been identified as a novel target of IL23 signaling and inhibition of CLDN8 could destroy the intestinal barrier function (Wang et al., 2016; Li et al., 2020). However, the roles of *CYP2C18*, *DPP10-AS1*, *CKB*, and *CP* involved in the IBD associated syndrome has not been reported. Therefore, the top DEGs identified in this study, especially those had not been reported, were deemed to be worthy of further investigation.

### Gene Set Enrichment of the Differentially Expressed Genes and Immune Signatures Associated With UC

**FIGURE 3:**
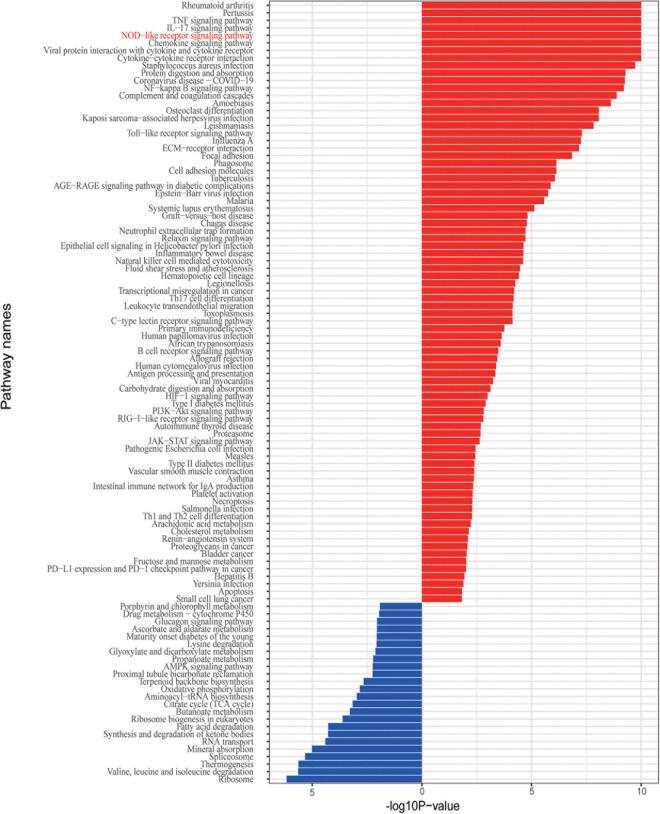
Enriched functional pathway associated with the DEGs.

Given that the IBD and related disorders are characterized by the dysregulated immune system, quality control based on gene set enrichment and pathway analysis were performed to check the confidence of the informative markers identified via DEG analysis. To estimate the underlying functional components, we performed gene set enrichment analysis and we found that the DEGs were significantly related to functions, such as ion response and chemokine activity (Figure 2C). To further investigate the dysfunction network underlying the pathogenesis of IBD, we performed functional pathway enrichment analysis. In our analysis, the top five enriched pathways were rheumatoid arthritis, pertussis, TNF signaling pathway, IL-17 pathway, and NOD-like receptor signaling pathway (Figure 3), which is consistent with the observation the NOD2-related pathways were involved in the pathogenesis mechanism underlying IBD. Generally, immune system dysregulation was highly enriched in the related functional pathways, and pathways related to virus infection recurred (Figure 3). Interestingly, we also found that the DEGs were also enriched in the Coronavirus disease (COVID-19) associated pathway, which was consistent with some recent publications (Allocca et al., 2020). Apart from the immune-related disorders, dysregulated expression of the identified DEGs might also be involved in other disorders, such as cancer. For instance, the PD1/PD-L1 checkpoint pathway was highly regulated among the affected twins (Figure 3). Therefore, individuals with IBDs may have a high predisposition to other disorders and might benefit from the immune checkpoint inhibitors treatment.

**FIGURE 4:**
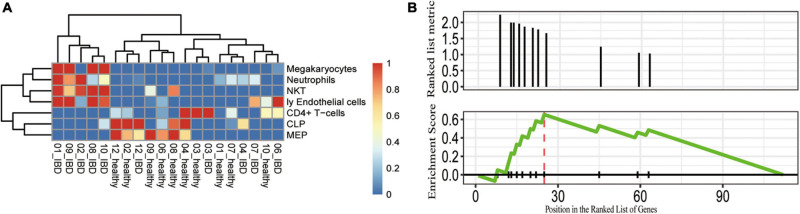
Potentially dysregulated immune cell subpopulations and pathways identified in the discordant IBD twin pairs. **(A)** The cell subpopulations that differ between the healthy and control. The scaled cell proportions were shown in the heatmap. **(B)** GSEA showing that the DEGs enriched in the gene set were upregulated in cortical and medullary thymocytes. CLP, common lymphoid progenitor; MEP, megakaryocytic-erythroid progenitors; Ly endothelial cells, lymphatic endothelial cells; NKT, natural killer T cells.

Given that abnormal immune responses were highly enriched in the IBDs, we further evaluated the dysregulated immune cell subpopulations. We applied xCell to estimate the immune cell subpopulation and we found seven immune cell subpopulations that were distinct between the affected and unaffected twins (Figure 4A). For instance, the proportion of megakaryocytes, neutrophils, natural killer T (NKT), and endothelial cells was highly increased in the UC affected twins; and the proportion of CD4^+^ T cell, CLP, and MEP subpopulations was significantly decreased in the UC affected twins (Figure 4B). Furthermore, we performed a GSEA of the immune-related gene sets, we found that the DEGs were enriched in cortical and medullary thymocyte gene sets (*P* = 0.0016; *q* < 0.05), and the genes with the greatest contribution were *VNN1*, *CHI3L1*, *APOBEC1*, *CXCL2*, *CXCL6*, *IL1RN*, *CXCL3*, and *IFIT3*. Therefore, the genetic-independent pathways involved in the pathogenesis of discordant twin pairs of IBDs were similar to the dysfunction of genetic components, such as NOD2.

### Deconvolution of the Tissue Specificity of the DEGs and Refinement of Candidate Genes

**FIGURE 5:**
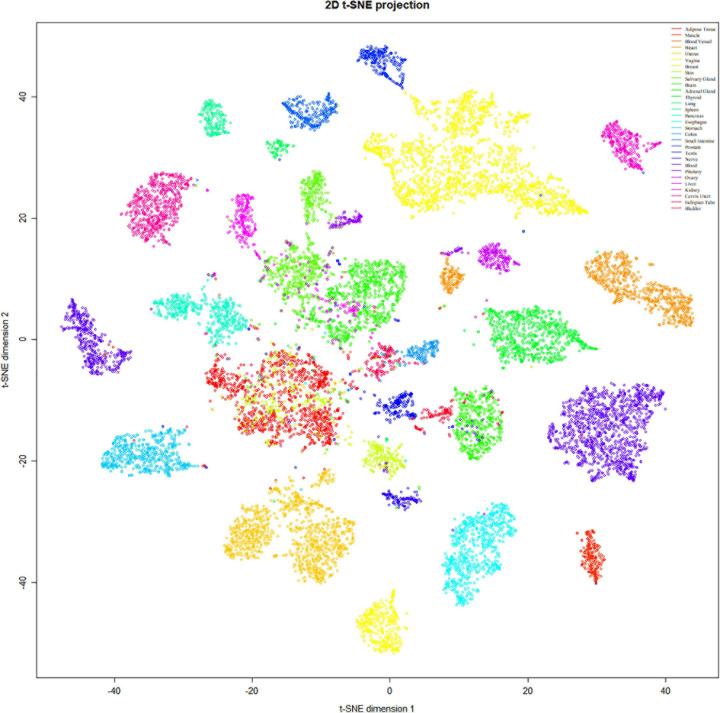
Tissue specificity of the DEGs was identified in the discordant IBD twin pairs. The expression spectrum across 30 different tissue types indicates a variable spectrum of the identified DEGs.

**FIGURE 6:**
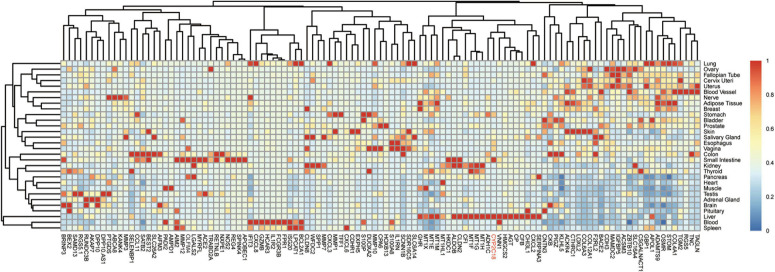
Expression spectrum of the DEGs across different tissues. Normalized expression values across the tissues were shown in the heatmap. Among the identified candidate genes for the pathogenesis of IBD, gene *CYP2C18* could be a novel marker for IBD, especially UC and *CYP2C18* was labeled in red.

**FIGURE 7:**
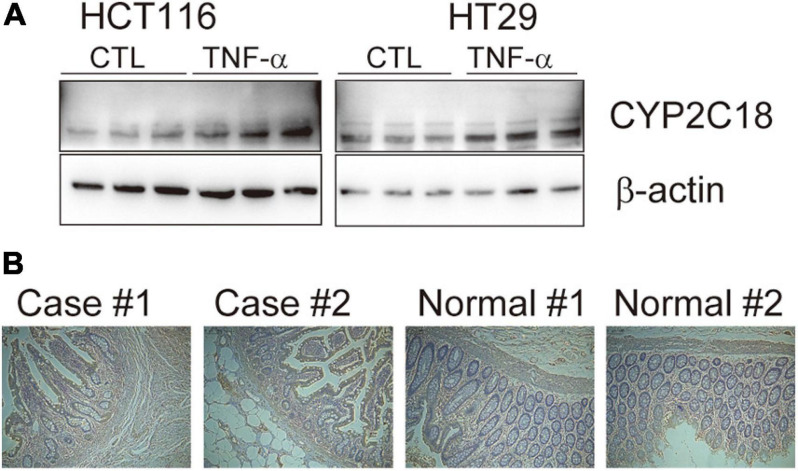
The expression level of *CYP2C18* was upregulated in colorectal cancer cell lines and UC-affected cases. **(A)** The expression level of CYP2C18 was highly increased after TNF-α stimulation. **(B)** Representative images of immunohistochemistry staining of CYP2C18 in UC affected cases and normal individuals. Scale bar: 100 μm.

To refine the candidate genes involved in IBD, we deconvolve the tissue expression specificity of the DEGs that do not have disease-causative supporting evidence in either GWAS FineMapping or DiseaseNet curation by t-SNE based approach. Due to *RP11-247L20.4*, *LOC283070*, *EMR2*, *GPR126*, *MFSD4*, and *GPR128* have unmatched gene id in the GTEx dataset, we rule them out from the downstream analysis. Based on the GTEx dataset, we found that those DEGs showed wide tissue specificity (Figure 5). After normalized the expression level, we found two clusters of genes, including *OLFM4*, *NXPE1*, *APOBEC1*, *CYP2C18* that were specifically expressed in either the colon or small intestine (Figure 6). Given that the role of the gene *CYP2C18* in the pathogenesis of IBDs has not been investigated, we mainly focused on determining the functional role of CYP2C18. Interestingly, we found multiple, rare, non-silent variants in *CYP2C18* (e.g., Tyr68Ter, Cys279Arg, and Thr243Met) that were enriched in IBD using the IBD exome browser (Supplementary Table 4). We performed functional assay to validate the results we observed. After inducing the inflammation signaling by TNF-α, the expression of *CYP2C18* was significantly increased in the HCT116 and HT29 cell lines (Figure 7A). Immunohistochemistry (IHC) analysis showed that the expression of CYP2C18 was highly increased in the UC-affected cases in comparison with the normal individuals (Figure 7B). Therefore, *CYP2C18* is a good candidate susceptible gene for IBDs and valuable for further investigation.

## Discussion

Shared etiological factors owing to both clinical and immunological characteristics among immune-mediated diseases (IMDs) have long been suspected (Parkes et al., 2013). Individuals who are affected by one type of IMD could have higher susceptibility to other IMDs. There are two main types of IMDs, seropositive autoimmune diseases, such as rheumatoid arthritis, and seronegative auto-inflammatory disorders, such as CD and psoriasis (Parkes et al., 2013). In our studies, we found that genes upregulated in UC are also enriched in rheumatoid arthritis and psoriasis. Therefore, IMDs could have some shared susceptibility loci and similar underlying genetic etiologies.

Consistent with the phenotypes observed in IBD, the DEGs identified in the discordant twin pairs were significantly enriched in the immune-related pathways and components. In our analysis, we found that the proportions of four immune-related cell subpopulations were increased in UCs. The proportion of megakaryocytes was significantly increased in the UC-affected twins, but the proportion of megakaryocytic-erythroid progenitor cells was decreased in the UC-affected twins. Megakaryocytes are derived from hematopoietic stem cell precursor cells in the bone marrow and produce platelets. Inflammation and coagulation are involved in the pathogenesis and clinical manifestation of IBDs (Voudoukis et al., 2014), and the increased influx of progenitor cells in the megakaryocyte cell compartment in IBD would alter the platelet count (Voudoukis et al., 2014). Interestingly, infliximab treatment significantly rescued pro-platelet formation by megakaryocytes from IBD patients but not by megakaryocytes from healthy controls (Di Sabatino et al., 2016), which confirms the specific role of megakaryocytes in the pathogenesis of IBDs.

Neutrophils have a dual role in intestinal inflammation, and overactivated neutrophils cause chronic inflammation in IBDs (Fournier and Parkos, 2012). NKT cells are involved in both the innate and adaptive immune responses and recognize CD1−restricted microbial and self-lipids, which participate in the development of IBD by regulating intestinal homeostasis (Lai et al., 2019; Brailey et al., 2020). In addition, during inflammation, the lymphatic system undergoes intense expansion through lymphangiogenesis, and aberrant increases in the lymphatic vasculature have been reported in patients with CD and UC (Danese, 2011). Therefore, the distinct proportions of immune cell subpopulations in UC-affected and unaffected twins explain, in part, the underlying pathogenesis of IBD.

By combining the expression features identified in our analysis with the rare variants enriched in IBD-affected twins, we identified some candidate makers for IBDs. Although we excluded random expression noise by including unrelated healthy twin pairs in our analysis, the sample size was quite small. In our analysis, the role of the top DEGs, such as *OLFM4*, *LOXL2*, *COL12A1*, *MMP1*, and *CLDN8*, involved in the pathogenesis of IBD has recently been identified. Gene *OLFM4* acts as a stem cell marker of the small intestine and is overexpressed in the inflamed colonic epithelium, especially in UC-affected cases. And we also identified some novel candidates which might have disease contributing roles. For instance, we observed that the expression of *CYP2C18* is highly upregulated in IBD-affected twins, and multiple rare non-silent variants in *CYP2C18* are enriched in IBDs. So far, the roles of *CYP2C18* in the pathogenesis of IBDs have not been elucidated. Therefore, we focused on validating the association between *CYP2C18* and UC.

*CYP2C18* encodes a member of cytochrome P450 (CYPs) superfamily of enzyme, which catalyze the metabolism of endogenous and exogenous substances, and extrahepatic and extrarenal CYP enzymes play critical roles in the development of UC (Sen and Stark, 2019). *In vitro*, the expression of *CYP2C18* was significantly upregulated in TNF-α induced inflammation status (Figure 7A). And the expression of CYP2C18 was also increased in the UC-affected case (Figure 7B). Based on the mouse model, various components such as P450 substrates and P450 metabolites in the blood may change with the UC-specific pattern (Yamamoto et al., 2018). The secretion of CYP2C18 in serum and varied expression of CYP2C18 between UC affected cases and UC unaffected cases, provide the possibility to evaluate UC using P450 enzyme through non-invasive method. However, the sample size was limited in our analysis, further investigation is needed to confirm our results and determine the detailed mechanism by which the identified genes contribute to the pathogenesis of IBDs.

## Data Availability Statement

The original contributions presented in the study are included in the article/Supplementary Material, further inquiries can be directed to the corresponding author/s.

## Ethics Statement

The colon tissues were collected with informed consent from UC affected cases and controls who had undergone surgery resection at the First Affiliated hospital of Zhejiang University, School of Medicine. Researches were performed under a protocol approved by Clinical Research Committee of the First Affiliated Hospitical, Zhejiang University School of Medicine (2020-593).

## Author Contributions

JD and JZ: conception and design. JD: administrative support. JD, JY, HD, and JZ: provision of study materials or patients. JY, HD, and JZ: collection and assembly of data. JD and JY: data analysis and interpretation. All authors wrote and approved the final manuscript.

## Conflict of Interest

The authors declare that the research was conducted in the absence of any commercial or financial relationships that could be construed as a potential conflict of interest.

## Footnotes

1 https://CRAN.R-project.org/package=factoextra

2 https://dmz-ibd.broadinstitute.org

## Supplementary Material

Supplementary Figure 1 Outlines of the analysis approaches involved in this study.

Supplementary Figure 2 Principle component analysis of healthy twin pairs. a and b indicate the matched individuals in each twin pairs.

Supplementary Figure 3 Volcano plot of the differentially expressed genes in the healthy twin pairs. All the genes show stable expression spectrum between the healthy twin pairs.

Supplementary Figure 4 Venn diagram of the candidate disease-causative genes with evidence from GWAS-FineMapped, DiseaseNet and the DEGs.

Supplementary Table 1 The enriched human phenotypes and disorders related to the fine-mapped genes.

Supplementary Table 2 Differentially expressed genes identified in the discordant IBD twin pairs. The adjusted *p*-value was computed by Benjamini-Hochberg correction.

Supplementary Table 3 The intersection of the genes across FineMapped, DiseaseNet, and DEGs.

Supplementary Table 4 The non-silent variants of CYP2C18 that were enriched in IBDs.
