# Supplementary figures and images for: Revisiting an Expression Dataset of Discordant Inflammatory Bowel Disease Twin Pairs Using a Mutation Burden Test Reveals CYP2C18 as a Novel Marker
Source: Front Genet. 2021 Jun 15;12:680125. doi: 10.3389/fgene.2021.680125 (PMC8239360; doi:10.3389/fgene.2021.680125)

Individuals – PCA

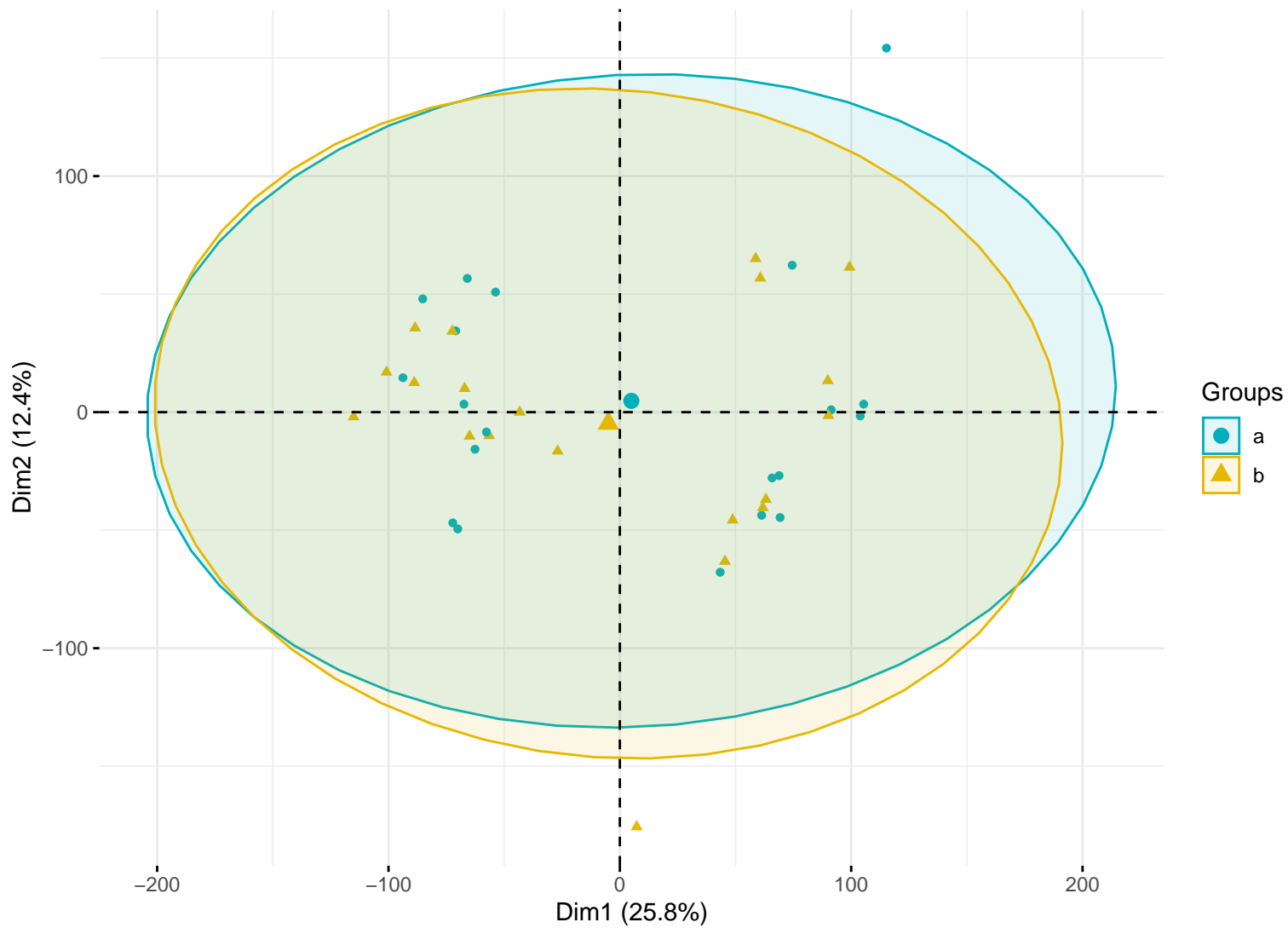

Supplement: Supplementary Figure 1 — Outlines of the analysis approaches involved in this study. [file Data_Sheet_1.zip › Figure S1.PDF]

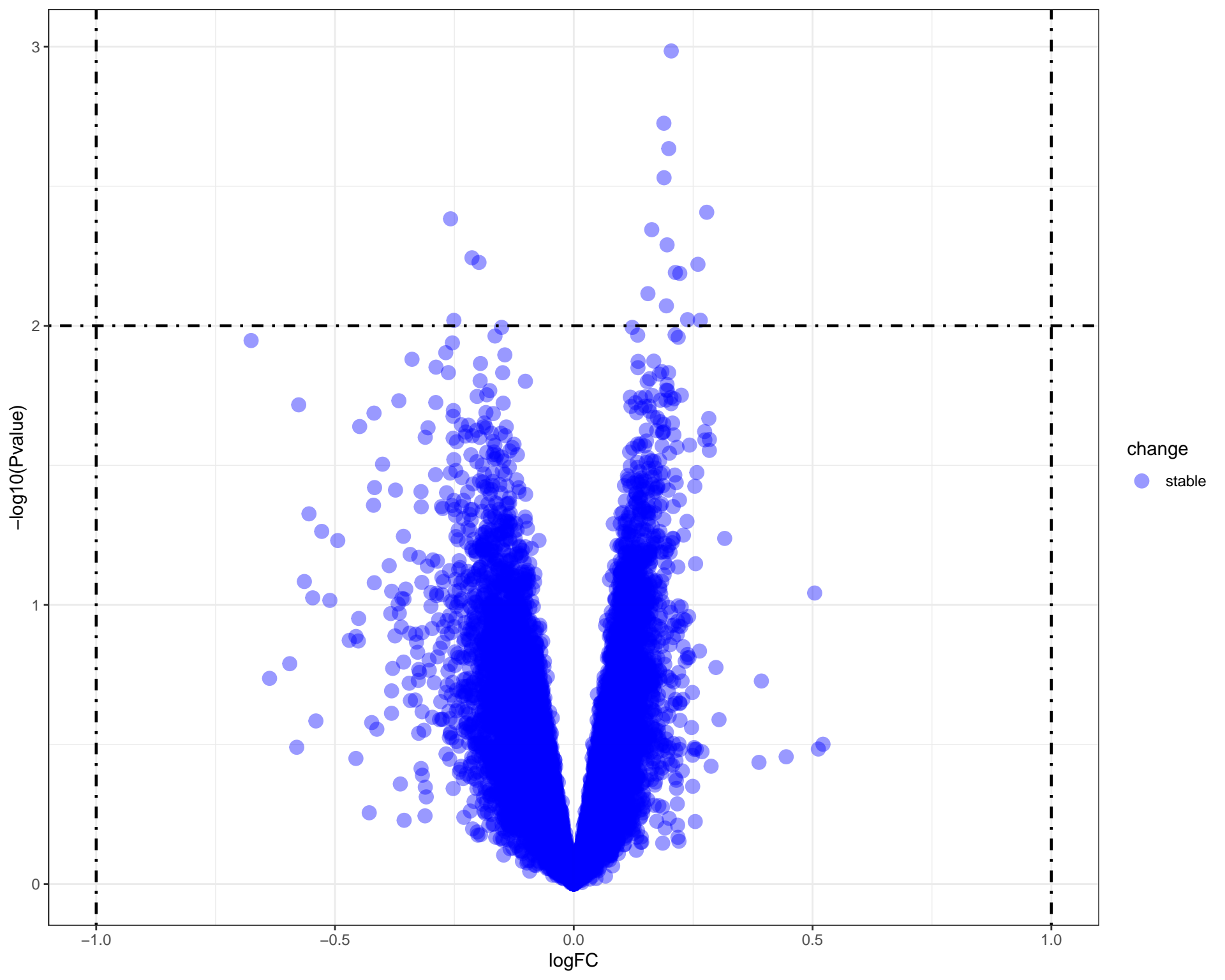

Supplement: Supplementary Figure 1 — Outlines of the analysis approaches involved in this study. [file Data_Sheet_1.zip › Figure S2.PDF]

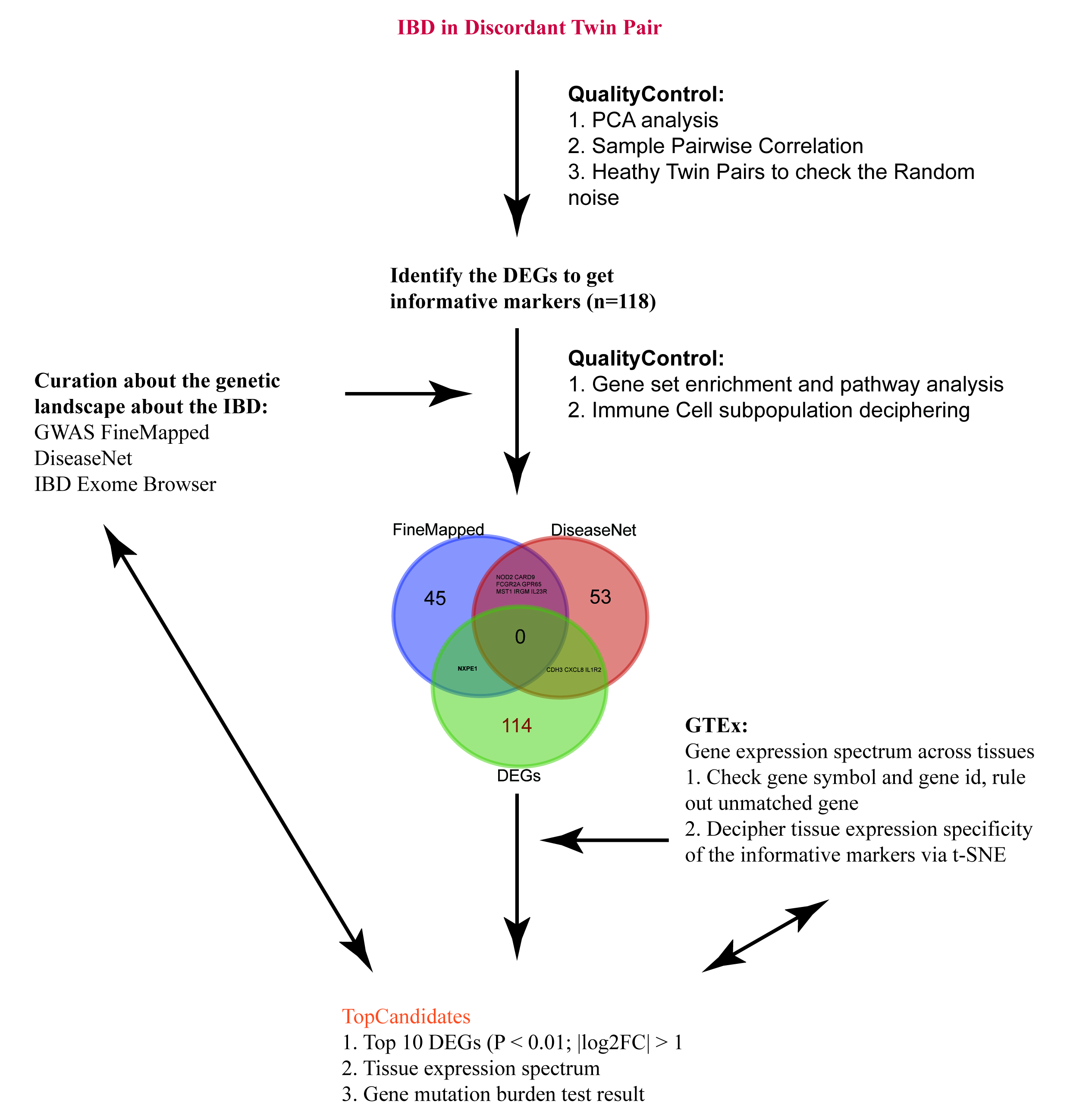

Supplement: Supplementary Figure 1 — Outlines of the analysis approaches involved in this study. [file Data_Sheet_1.zip › Figure S3.TIF]

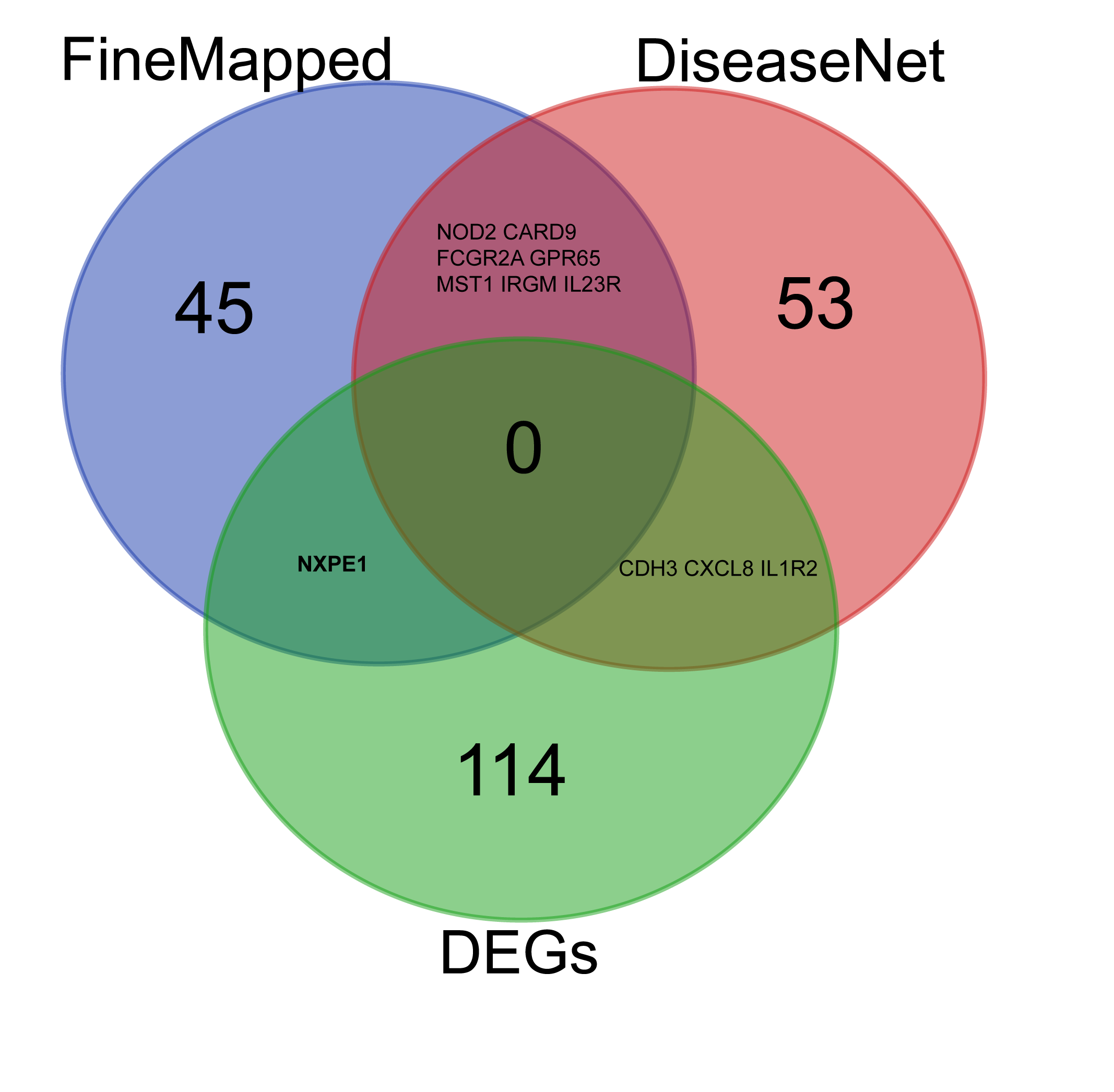

Supplement: Supplementary Figure 1 — Outlines of the analysis approaches involved in this study. [file Data_Sheet_1.zip › Figure S4.TIF]
